# Latent profile analysis of change fatigue among clinical nurses: a cross-sectional study

**DOI:** 10.3389/fpubh.2026.1779473

**Published:** 2026-05-13

**Authors:** Xiangwen You, Haibo Ma, Rong Yan, Miao Yao, Hongyan Lu

**Affiliations:** 1 General Hospital of Ningxia Medical University, Yinchuan, China; 2 Ningxia Medical University, Yinchuan, China

**Keywords:** change fatigue, clinical nurses, cross-sectional study, latent profile analysis, work engagement

## Abstract

**Aim:**

To identify latent subgroups of change fatigue among clinical nurses and explore their associations with work engagement.

**Methods:**

Between February and May 2025, clinical nurses from 13 public hospitals in Ningxia Hui Autonomous Region, northwestern China, were enrolled using convenience sampling. Data were gathered using a sociodemographic questionnaire, the Chinese version of the Change Fatigue Scale, and the Chinese version of the Utrecht Work Engagement Scale. All analyses were completed in SPSS 26.0 and Mplus 8.1. Latent profile analysis was used to analyze their change fatigue subgroups. The R3STEP method was applied to identify predictors of profile membership, and the BCH method was employed to compare differences in work engagement across subgroups.

**Results:**

Of the 2,482 participants, the total score of change fatigue was 26.67 ± 9.97. Three latent subgroups of change fatigue were identified: ‘Low change fatigue group’ (24.21%), ‘Moderate change fatigue group’ (46.62%), and ‘High change fatigue group’ (29.17%). The distribution of these profiles was significantly associated with years of service, hospital grade, monthly income and change exposure. Moreover, these change fatigue profiles were negatively associated with nurse work engagement. Specifically, work engagement was significantly higher in the low change fatigue group than in the moderate and high groups, with no significant difference between the moderate and high groups.

**Conclusion:**

Clinical nurses exhibited three distinct change fatigue profiles, highlighting substantial heterogeneity. Years of service, hospital grade, monthly income and change exposure were key demographic predictors of profile membership. The study demonstrates that different change fatigue profiles were significantly associated with work engagement of clinical nurses, suggesting that reducing nurses’ change fatigue is essential for improving their work engagement.

## Introduction

Change fatigue refers to a state in which individuals are exposed to constant organizational changes. It manifests as exhaustion, diminished agency, and passive acceptance of change (1). It is often regarded as a critical barrier to the effective implementation of organizational change initiatives (2). With the rapid evolution of the healthcare environment, organizational change occurs frequently in healthcare settings to meet the patient’s increasing demand for personalized and high-quality services (3, 4). These changes encompass technology and equipment upgrades, workflow adjustments, infrastructure improvements, human resources realignments and other facets (5, 6). However, such frequent and continuous changes can elicit substantial stress among clinical nurses, who play a key role in successfully implementing organizational change (7, 8). Specifically, nurses are challenged to adapt to revised work procedures, cope with elevated workloads, undergo role reorientation, endure job uncertainty, and navigate inadequate communication and support during organizational transitions (9).

Change fatigue is often subtle, insidious and passive in nature, which allows its adverse consequences to remain unrecognized (10). As documented in nursing research, long-term change fatigue exerts detrimental effects on nurses at both individual and organizational levels (11). At the individual level, the persistent effort to adapt to continuous new changes without sufficient time or support aggravates occupational stress, leading to burnout, and reduced job satisfaction (12). At the organizational level, change fatigue may hinder the rollout of new initiatives, raise the risk of nursing errors, and undermine care quality and team stability (13). In summary, change fatigue constitutes a critical concern in clinical nursing settings and merits increased attention.

Within the nursing discipline, research interest in change fatigue has been steadily rising globally. Research indicated that nurses are approximately three times more likely to experience change fatigue than other healthcare professionals (14). McMillan’s study highlighted that nurses experience the core dimensions of change fatigue, including emotional exhaustion, apathy, powerlessness, and burnout (15). A survey conducted in the USA found that nurses reported moderate levels of change fatigue (10). This issue appears even more pronounced among Chinese nurses. A survey of 451 clinical nurses from a tertiary general hospital in China documented moderate levels of change fatigue (7), while another Chinese cross-sectional study further revealed that nurses experienced moderate-to-high levels of change fatigue (5). Previous studies have mostly focused on investigating the total score of change fatigue through a variable-centered approach, ignoring individual variations and potential differences (5, 7, 10). Latent profile analysis (LPA) is a person-centered method that can classify participants into distinct latent profiles according to their responses to measured variables (16, 17). Existing research on the heterogeneity of change fatigue among clinical nurses is limited; only Ma et al.’s study conducted in coastal cities of China has explored this heterogeneity, finding that nurses’ change fatigue can be classified into three latent profiles (18). Furthermore, there is a lack of research examining the relationship between change fatigue profiles and work engagement among clinical nurses. This gap highlights the need for research that identifies the heterogeneity of change fatigue and its associations with work engagement.

Work engagement represents a positive, fulfilling, work-related state of mind characterized by vigor, dedication, and absorption (19). Nurses with high work engagement are more inclined to provide optimal high-quality care and contribute to enhanced patient outcomes. Moreover, such work engagement promotes nurses’ occupational well-being, mitigates nursing turnover, and strengthens care continuity, thereby facilitating the formation of stable nursing teams (20). The conservation of resources (COR) theory posits that individuals have an innate motivation to acquire, retain, protect, and foster their valued personal resources, and stress reactions occur when there is an actual or perceived loss of these resources (21). Change fatigue can be viewed as a state of resource depletion caused by continuous and demanding organizational changes. For clinical nurses, high levels of change fatigue entail the continuous consumption of their emotional, cognitive, and physical resources without sufficient compensation. Given that work engagement heavily relies on the investment of abundant personal resources, such resource conservation behaviors may further influence their work engagement. Previous studies have shown that change fatigue and work engagement are closely associated, with burnout playing a mediating role (22). This finding was based on a sample of 199 general working adults, which may limit its generalizability to the nursing population. In the context of clinical nursing, exploring the relationship between change fatigue and work engagement is necessary. Moreover, research on the specific profiles of change fatigue among clinical nurses and their association with nurses’ work engagement remains limited.

Therefore, this study aims to identify different profiles of change fatigue among clinical nurses, thereby revealing their inherent heterogeneity. We further examine the association between different change fatigue profiles and work engagement. These findings can serve as a theoretical basis for developing personalized interventions and promoting nurses’ work engagement.

Based on the above content, this study proposes the following hypotheses: (1) Clinical nurses exhibit distinct latent profiles of change fatigue; (2) Sociodemographic and organizational factors significantly predict profile membership; (3) Work engagement differs significantly across distinct change fatigue profiles.

## Methods

### Study design and participants

A cross-sectional, descriptive exploratory design was adopted. Participants were recruited between February and May 2025 across 13 public hospitals in Ningxia Hui Autonomous Region, China, using the convenience sampling method. Inclusion criteria were as follows: (a) registered nurses who worked in clinical frontline, (b) a minimum of 1 year of clinical experience, and (c) signed informed consent. The exclusion criteria were (a) nurses who came to the hospital for further study and training, (b) nurses who were not on duty for various reasons (such as further training, vacation, and health issues). Considering nurses with at least 1 year of clinical experience have fully adapted to clinical work, accumulated certain practical experience in dealing with daily work challenges and organizational changes, and can more comprehensively perceive the impact of organizational changes on their work status and psychological state. According to Kendall’s criteria of sample estimation, the sample size is 10 times the number of scale items. In this study, a total of 27 items were included. Therefore, the required minimal sample size was *N* = 10 × 27 = 270. Considering an invalid response rate of 20% during the study, the final sample size required was *N* = 270 ÷ (1–20%) ≈ 338. As suggested by previous studies, LPA generally requires a sample size larger than 500 (23). A total of 2,686 questionnaires were collected in this study, after excluding 204 responses that took less than 180 s to complete, 2,482 valid questionnaires were retained, yielding an effective response rate of 92.41%.

### Instruments

#### The sociodemographic characteristics questionnaire

A sociodemographic questionnaire was developed based on the literature review. It collected participant characteristics, which included age, gender, educational attainment, marital status, working years, professional title, grade of hospital, mode of appointment, monthly income (in CNY), shift system, specialty nurses, and whether exposure to frequent or continuous organizational changes.

#### The Chinese version of change fatigue scale (CFS)

The Change Fatigue Scale was administered to assess change fatigue levels in clinical nurses (24). This unidimensional instrument comprises six items rated on a 7-point Likert scale (1 = strongly disagree to 7 = strongly agree), yielding total scores ranging from 6 to 42. Higher scores indicate more severe change fatigue. The scale demonstrated strong reliability with Cronbach’s *α* coefficients of 0.85 in non-nurse populations, 0.918 in Zhang et al.’s (25) Chinese adaptation, and 0.939 in the current study.

#### The Chinese version of the Work Engagement Scale-9 (UWES-9)

Work engagement was measured using the Chinese version of the Utrecht Work Engagement Scale-9 (UWES-9), originally developed by Schaufeli and Bakker (19) and cross-culturally adapted by Fong et al. for Chinese populations (26). The 9-item instrument assesses three dimensions: vigor, dedication, and absorption. Items are scored on a 7-point Likert scale (0 = never to 6 = daily), with total scores ranging from 0 to 54. Higher scores reflect greater work engagement. In this study, the scale achieved a Cronbach’s *α* of 0.936.

### Data collection

The project leader contacted Director of the Nursing Department at 13 public hospitals via telephone to explain the study’s purpose, methodology, and significance, obtaining institutional consent. Clinical nurses were recruited through convenience sampling. Eligible participants received electronic survey invitations distributed via hospital nursing departments. Data collection utilized Wenjuanxing (https://www.wjx.cn), a secure online survey platform in China. To ensure data integrity: all items were mandatory; IP address restrictions prevented duplicate submissions; a minimum completion time of 180 s was enforced. After a 4-month collection period, surveys were closed following 3 consecutive days without new submissions. Two researchers exported raw data and screened questionnaires using these exclusion criteria: identical responses throughout; logical inconsistencies; completion time <180 s.

### Statistical analysis

Data descriptive analyses were performed using SPSS 26.0. Continuous variables conforming to a normal distribution were reported as mean ± standard deviation, whereas categorical and ordinal variables were described using frequencies and percentages. Prior to LPA, six items of the Change Fatigue Scale were standardized using Z-score transformation. Specifically, each item was standardized to a mean of 0 and a standard deviation of 1 to eliminate the influence of different item scales on profile identification. LPA was then conducted using Mplus 8.1 to identify distinct subgroups of change fatigue among clinical nurses. A robust maximum likelihood (MLR) estimator was applied for model estimation. To ensure the stability of the model solution and avoid local optima, 1,300 random starting sets and 250 final-stage optimizations (STARTS = 1,300 250) were used. Models specifying one to four latent profiles were sequentially estimated, starting with a one-profile solution. Model fit was assessed using the Akaike Information Criterion (AIC), the Bayesian Information Criterion (BIC), and the sample-size adjusted BIC (aBIC), with lower values indicating better fit. Classification accuracy was evaluated using entropy (range: 0–1; values closer to 1 preferred). The Lo–Mendell–Rubin (LMR) and Bootstrapped Likelihood Ratio Test (BLRT) were employed to determine whether a k-class model fit better than a model with k-1 classes, and a significant *p*-value (*p* < 0.05) indicated that the k-class model provided a superior fit compared to the k-1-class model (27, 28). The minimum proportion of each latent profile was maintained at no less than 5% to ensure interpretability. To determine the optimal number of profiles, the best-fitting model was selected based on both statistical fit indices and substantive interpretation.

The R3STEP procedure in Mplus was conducted to examine the associations between demographic and occupational characteristics and latent change fatigue profile membership. The BCH command in Mplus was applied to examine the outcome variable of work engagement. This approach enabled across-profile comparisons and statistically tested whether distinct latent profiles differed significantly in their levels of work engagement (29). The advantages of R3STEP and BCH are that they enhance statistical power by accounting for classification uncertainty.

### Ethical considerations

This cross-sectional study was conducted in accordance with the principles of the Declaration of Helsinki. It was approved by the Ethics Review Committee of the General Hospital of Ningxia Medical University (Approval No.: KYLL-2025-1371). All participants provided informed consent through the online platform before completing the questionnaire. The first page of the online questionnaire was presented in consistent language to clarify the study’s purpose and methodology. Participants were informed that their participation was voluntary and anonymous, with the right to withdraw at any time. Data were collected and analyzed anonymously to protect participants’ privacy and confidentiality, and no identifying information was included in the dataset.

## Results

### Sociodemographic characteristics of participants

A total of 2,482 participants were included in the analysis. They had a mean age of 35.95 ± 6.33 years, with ages ranging from 21 to 59 years. Among them, 97.1% were female (*n* = 2,409), 78.9% were aged ≤ 40 years (*n* = 1,957), 88.2% were married (*n* = 2,189), 90.1% held a bachelor’s degree or above (*n* = 2,237), and 27.3% (*n* = 678) self-reported being exposed to frequent or continuous organizational change. 851 nurses (34.3%) were from internal medicine wards, 568 nurses (22.9%) from surgical wards, 182 nurses (7.3%) from the intensive care unit (ICU), and 881 nurses (35.5%) from other clinical departments. Regarding work experience, more than half of the participants (50.9%, *n* = 1,263) had between 11 and 20 years. Demographic characteristics are detailed in Table 1.

**Table 1:** General characteristics of participants (*N* = 2,482).

Variable	Classification	All samples	C1	C2	C3
		*n* (*%*)	*n* (*%*)	*n* (*%*)	*n* (*%*)
Hospital grade	3A	1,565 (63.0)	399 (66.4)	731 (63.2)	435 (60.1)
	3B	535 (21.6)	121 (20.1)	264 (22.8)	150 (20.7)
	2A	382 (15.4)	81 (13.5)	162 (14.0)	139 (19.2)
Gender	Female	2,409 (97.1)	582 (96.8)	1,125 (97.2)	702 (97.0)
	Male	73 (2.9)	19 (3.2)	32 (2.8)	22 (3.0)
Marital status	Unmarried	216 (8.7)	48 (8.0)	102 (8.8)	66 (9.1)
	Married	2,189 (88.2)	536 (89.2)	1,018 (88.0)	635 (87.7)
	Divorced/others	77 (3.1)	17 (2.8)	37 (3.2)	23 (3.2)
Age (years)	21–30	488 (19.7)	144 (24.0)	209 (18.1)	135 (18.7)
	31–40	1,469 (59.2)	350 (58.2)	683 (59.0)	436 (60.2)
	> 40	525 (21.1)	107 (17.8)	265 (22.9)	153 (21.1)
Mean age		35.95 ± 6.33	35.34 ± 6.22	36.23 ± 6.41	36.00 ± 6.28
Years of service	2–10	868 (35.0)	241 (40.1)	395 (34.1)	232 (32.0)
	11–20	1,263 (50.9)	287 (47.8)	591 (51.1)	385 (53.2)
	> 20	351 (14.1)	73 (12.1)	171 (14.8)	107 (14.8)
Mean years of service		13.73 ± 6.82	13.18 ± 6.73	13.87 ± 6.87	13.94 ± 6.82
Education level	Bachelor degree	2,230 (89.8)	539 (89.7)	1,037 (89.7)	654 (90.4)
	Master degree	7 (0.3)	1 (0.2)	5 (0.4)	1 (0.1)
	Without degree	245 (9.9)	61 (10.1)	115 (9.9)	69 (9.5)
Professional title	Staff nurse	128 (5.2)	38 (6.3)	51 (4.4)	39 (5.4)
	Senior nurse	1,040 (41.9)	273 (45.4)	468 (40.4)	299 (41.3)
	Charge nurse	1,140 (45.9)	255 (42.4)	546 (47.2)	339 (46.8)
	Deputy chief nurse	149 (6.0)	32 (5.3)	82 (7.1)	35 (4.8)
	Chief nurse	25 (1.0)	3 (0.5)	10 (0.9)	12 (1.7)
Employment type	Tenured	349 (14.1)	67 (11.1)	173 (15.0)	109 (15.1)
	Contract	1908 (76.8)	489 (81.4)	878 (75.8)	541 (74.7)
	Agency staff	225 (9.1)	45 (7.5)	106 (9.2)	74 (10.2)
Monthly income (yuan)	< 5,000	627 (25.3)	138 (23.0)	274 (23.7)	215 (29.7)
	5,000–10,000	1768 (71.2)	453 (75.3)	836 (72.3)	479 (66.2)
	> 10,000	87 (3.5)	10 (1.7)	47 (4.0)	30 (4.1)
Shift situation	3 shift system	1,637 (66.0)	375 (62.4)	770 (66.6)	492 (68.0)
	2 shift system	845 (34.0)	226 (37.6)	387 (33.4)	232 (32.0)
Specialty nurses	Yes	565 (22.8)	131 (23.2)	280 (24.2)	154 (21.3)
	No	1917 (77.2)	470 (78.2)	877 (75.8)	570 (78.7)
EFCOC	Yes	678 (27.3)	74 (12.3)	314 (27.1)	290 (40.1)
	No	1804 (72.7)	527 (87.7)	843 (72.9)	434 (59.9)

C1, low change fatigue group; C2, moderate change fatigue group; C3, high change fatigue group; 3A, Grade 3 Class A hospital; 3B, Grade 3 Class B hospital; 2A, Grade 2 Class A hospital; yuan is the basic unit of Renminbi (RMB), the currency of China; EFCOC, exposure to frequent or continuous organizational change.

### Latent profiles analysis results of change fatigue among clinical nurses

The total score of change fatigue among clinical nurses was (26.67 ± 9.97) points. LPA was conducted on the change fatigue of 2,482 clinical nurses by sequentially constructing models with one to five latent profiles. The optimal solution was the 3-profile model based on a comprehensive evaluation of the latent profile indicators. As shown in Table 2, AIC, BIC, and aBIC decreased continuously from the 1-profile to 3-profile model, and the decline magnitude attenuated sharply after the 3-profile model, indicating a clear inflection point. Although both LMRT and BLRT remained significant for the 4-profile solution, the 4-profile model provided no substantial improvement in model fit. In addition, the minimum proportion of the 4-profile models was 10.44%, suggesting that this category was not representative. In contrast, the 3-profile model showed high entropy (0.931), suggesting excellent classification accuracy. All three profiles accounted for adequate proportions (24.21, 46.62, 29.17%) without underrepresented subgroups. Considering both statistical fit and theoretical interpretability, the 3-profile model was determined as the optimal model. To evaluate the classification accuracy and reliability of the latent profile analysis results, the average posterior probabilities for each of the three profiles were calculated. The results showed that the average probabilities of correct classification ranged from 96.8 to 97.4%, suggesting that the model results with three profiles were reliable (see Table 3).

**Table 2:** Comparison of fitting parameter indexes for various latent profile models.

Model	Loglikelihood	AIC	BIC	aBIC	*p-*value		Entropy	Category probability (%)
					LMR	BLRT		
1	−30667.679	61359.358	61429.160	61391.033	*NA*	*NA*	*NA*	100
2	−26706.247	53450.493	53561.013	53500.645	< 0.001	< 0.001	0.908	32.72/67.28
3	−24415.716	48883.433	49034.670	48952.062	< 0.001	< 0.001	0.931	24.21/46.62/29.17
4	−23646.146	47358.291	47550.246	47445.397	< 0.001	< 0.001	0.942	10.44/28.81/18.25/42.51

AIC, Akaike Information Criterion; BIC, Bayesian Information Criterion; a BIC, Sample-Adjusted Bayesian Information Criterion; Likelihood Ratio Test; Entropy, information entropy; LMR, Likelihood Ratio Test; BLRT, Bootstrap Likelihood Ratio Test.

**Table 3:** Average attribution probabilities for each latent profile.

Model	C1	C2	C3
C1	0.968	0.032	0
C2	0.016	0.966	0.017
C3	0	0.026	0.974

C1, low change fatigue group; C2, moderate change fatigue group; C3, high change fatigue group.

### Naming of latent categories for change fatigue among clinical nurses

**Figure 1:**
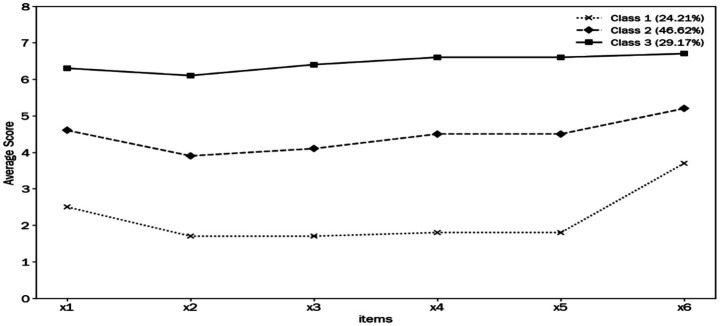
Latent profile characteristics of change fatigue.

A profile plot was constructed to illustrate the score distribution of the six items on the change fatigue scale among the three identified latent classes. The horizontal axis denotes the item order of the change fatigue scale, while the vertical axis denotes the standardized item score (see Figure 1). In accordance with the LPA results and the score characteristics shown in the profile plot, the three latent classes were assigned distinct labels. The three latent profiles were characterized as follows: Profile 1 (C1) accounted for 24.21% of the participants (*n* = 601). This profile exhibited the lowest mean scores across all change fatigue items, with an overall mean score of 12.92 ± 4.92, and was therefore designated as the ‘Low change fatigue group’. Profile 2 (C2) constituted the largest proportion of the sample (46.62%, *n* = 1,157), characterized by consistently moderate mean scores across all items and an overall mean change fatigue score of 26.60 ± 3.49, thus named the ‘Moderate change fatigue group’. Profile 3 (C3) comprised 29.17% of the participants (*n* = 724); this profile demonstrated the highest mean scores across all items, with an overall mean change fatigue score of 38.21 ± 3.35, and was therefore classified as the ‘High Change Fatigue Group’.

### Predictive variables for change fatigue profiles

The results of the R3STEP method for predicting variables are shown in Table 4. The findings indicate that years of service, hospital grade, monthly income and change exposure were significantly associated with nurses’ latent profile membership of change fatigue, with the ‘Low change fatigue group’ as the reference group. Compared to the ‘Low change fatigue group’, nurses with longer working years are 1.016 times more likely to be classified as ‘Moderate change fatigue group’ and 1.017 times more likely to be classified as ‘High change fatigue group’. This finding suggests that clinical nurses with longer professional tenure are more prone to elevated levels of change fatigue. Moreover, compared with the reference group, nurses in Grade 3A hospitals exhibited a significantly reduced probability of being classified into the ‘High change fatigue group’ (OR = 0.628, *p* < 0.05). Additionally, nurses with a monthly income of 5,000–10,000 yuan exhibited a significantly lower risk of membership in the ‘High change fatigue group’ (OR = 0.672, *p* < 0.05), and nurses with a monthly income over 10,000 yuan had a significantly higher likelihood of being categorized into the ‘Moderate change fatigue group’ (OR = 2.520, *p* < 0.05). Lastly, compared to nurses who have not experienced frequent or continuous organizational changes, those who have experienced frequent or continuous organizational changes are 5.054 times more likely to be classified into the ‘High change fatigue group’ (*p* < 0.001). Similarly, higher change exposure increased nurses’ likelihood of being categorized into the ‘Moderate change fatigue group’ by 2.780 times relative to the reference group (*p* < 0.001).

**Table 4:** Results of multinomial logistic regressions for the effects of predictors on Profile Membership (R3STEP).

Predictor variables	Moderate change fatigue group			High change fatigue group		
	b (SE)	OR	95% CI	b (SE)	OR	95% CI
Years of service	0.016 (0.008)	1.016*	1.000, 1.033	0.017(0.009)	1.017*	1.001, 1.035
Hospital grade						
3A	−0.084 (0.159)	0.919	0.674, 1.255	−0.465 (0.161)	0.628*	0.459, 0.860
3B	0.100 (0.185)	1.106	0.769, 1.590	−0.332 (0.191)	0.718	0.493, 1.044
2A (Ref.)						
Monthly income (yuan)						
> 10,000	0.924 (0.397)	2.520*	1.156, 5.492	0.698 (0.407)	2.011	0.906, 4.463
5,000–10,000	−0.070 (0.127)	0.932	0.727, 1.195	−0.398 (0.131)	0.672*	0.520, 0.868
< 5,000 (Ref.)						
EFCOC						
Yes	1.022 (0.153)	2.780***	2.062, 3.748	1.620 (0.154)	5.054***	3.739, 6.833
No (Ref.)						

Reference group, low change fatigue group; *p < 0.05; ***p < 0.001; yuan is the basic unit of Renminbi (RMB), the currency of China.

OR, odd ratios; SE, standard error of coefficient; 95% CI: 95% confidence interval; EFCOC, Exposure to frequent or continuous organizational change.

### Outcome variables of latent profiles of change fatigue

As presented in Table 5, after adjusting for years of service, hospital grade, and change exposure, the adjusted BCH analyses revealed significant differences in work engagement and its three dimensions across the three change fatigue profiles (all *p* < 0.001). Notably, nurses categorized as ‘Low change fatigue group’ demonstrated the highest levels of work engagement. Nurses within the ‘Moderate change fatigue group’ and ‘High change fatigue group’ exhibited comparable levels of work engagement, with no significant difference between them (*χ*^2^ = 1.021, *p* = 0.312). Post-hoc tests further confirmed that work engagement scores in the ‘Low change fatigue group’ were significantly higher than those in both the ‘Moderate change fatigue group’ (*χ*^2^ = 115.062, *p* < 0.001) and ‘High change fatigue group’ (*χ*^2^ = 106.734, *p* < 0.001).

**Table 5:** BCH results for the differences on nurses’ work engagement and three dimensions across latent profiles.

Variables	C1	C2	C3	Overall test	
	Mean (SE)	Mean (SE)	Mean (SE)	Adjusted BCH χ^2^	Pairwise comparison
Work engagement	50.750 (0.581)	43.083 (0.390)	42.374 (0.566)	140.267***	1 > 2, 1 > 3
Vigor	16.68 (0.198)	14.18 (0.13)	13.91 (0.19)	130.611***	1 > 2, 1 > 3
Dedication	17.13 (0.22)	14.25 (0.15)	13.78 (0.22)	147.129***	1 > 2, 1 > 3
Absorption	16.94 (0.20)	14.65 (0.14)	14.68 (0.19)	95.644***	1 > 2, 1 > 3

C1, low change fatigue group; C2, moderate change fatigue group; C3, high change fatigue group; ***p < 0.001.

## Discussion

### Current status of change fatigue among clinical nurses

With a mean total score of 26.67 ± 9.97 on the change fatigue scale (higher than the theoretical median of 24), the sample exhibited a moderately high severity of change fatigue. The result is significantly higher than the moderate levels reported by Yu et al. (7) and Brown et al. (11), however, it was lower than those reported by Lv et al. (5). This discrepancy may be attributed to differences in the study populations, as our study featured a larger sample size and broader sample sources. In the fast-paced healthcare environment, organizational changes have become increasingly prevalent (11, 30). As key members of the healthcare delivery system, clinical nurses are among the professional groups with the most frequent direct patient contact. They are often at the forefront of driving and implementing most change initiatives. Clinical nurses must balance routine duties with the ongoing pressure to adapt to successive changes (10). Therefore, the issue of change fatigue among clinical nurses merits urgent attention.

### Latent profiles of change fatigue among clinical nurse

Based on LPA with a large sample of clinical nurses, the current study identified three distinct latent profiles of change fatigue, categorized by scale item responses. This result is consistent with previous studies (13, 18). Low change fatigue group (C1): Representing 24.21% of the sample, this group exhibited the lowest level of change fatigue. Nurses in this profile were predominantly novices with relatively little exposure to organizational changes. They demonstrated a strong work ethic and were more likely to perceive change as energizing. Moderate change fatigue group (C2): Representing the largest proportion of nurses (46.62%), almost half of the sample fell into this profile, and this group showed a consistently moderate level of change fatigue across all items. High change fatigue group (C3): Comprising 29.17% of the nurses, this group reported significantly higher levels of change fatigue than both the C1 and C2 groups. In the present study, the ‘High change fatigue group’ had significantly higher scores than the ‘Moderate change fatigue group’ across all items, while the ‘Moderate change fatigue group’ scored higher than the ‘Low change fatigue group’. These findings indicate that the majority of clinical nurses experience moderate to high levels of change fatigue. Therefore, it is recommended that greater attention should be paid to nursing populations with moderate or higher levels of change fatigue to sustain the stability of the nursing workforce. Moreover, a notable finding was that Item 6 (“I would like to have a period of stability before the organization introduces any new change”) consistently scored markedly higher than other items across the three latent profiles. This finding provides clear empirical support for implementing buffer periods between sequential organizational change initiatives (31). Granting nurses sufficient time to adapt to and consolidate new workflows, protocols, or technologies before the introduction of further changes can effectively alleviate fatigue and reduce saturation effects caused by frequent, unrelenting reforms (32). Nursing managers should utilize a structured change calendar, a proactive planning instrument that monitors, appraises, and coordinates organizational initiatives on a consistent weekly or monthly schedule (33). Systematically mapping and strategically spacing out change initiatives can effectively lower the incidence of change fatigue and enhance organizational preparedness for future reforms (16, 34).

### Predictive variables for change fatigue profiles among clinical nurses

This study employed the R3STEP method to explore the predictive variables of latent change fatigue profiles among clinical nurses. The findings revealed that years of service, hospital grade, monthly income, and change exposure were significant predictors of nurses’ change fatigue profile membership. Firstly, the results indicated that nurses with longer years of service were more likely to fall into the moderate or high change fatigue groups. This suggests that long-term professional tenure may accumulate work-related stress and reduce adaptability to organizational changes. Clinical nurses with longer working years often bear more work responsibilities, witness repeated organizational adjustments, and face persistent physical and mental exhaustion, which gradually erodes their resilience to change and ultimately increases the risk of elevated change fatigue (35, 36). Conversely, novice nurses may have a stronger ability to adapt to new changes and a lower cumulative burden of change, thus being more likely to remain in the ‘Low change fatigue group’. Secondly, nurses in Grade 3A hospitals had a significantly reduced probability of being in the ‘High change fatigue group’. Grade 3A hospitals often have more scientific change management mechanisms, including adequate pre-change training, timely communication of change objectives, and effective support for nurses during the change process. These measures help reduce nurses’ uncertainty and resistance to change, thereby mitigating the risk of high change fatigue. This finding highlights the importance of improving hospital management capacity and optimizing change implementation strategies in reducing nurses’ change fatigue. Thirdly, monthly income showed a differential predictive effect on nurses’ change fatigue profiles. Specifically, nurses with a monthly income of 5,000–10,000 yuan had a significantly lower risk of being in the ‘High change fatigue group’, while those with a monthly income over 10,000 yuan were more likely to be classified into the ‘Moderate change fatigue group’. This interesting finding suggests that income level is not linearly related to change fatigue. Nurses with moderate income may have a better balance between work income and work pressure, which enhances their job satisfaction and reduces the negative impact of organizational changes. In contrast, nurses with high monthly income may bear higher work responsibilities and work intensity, and the associated work pressure may offset the protective effect of high income, making them more prone to moderate change fatigue (37). This result reminds us that improving income alone is not sufficient to alleviate change fatigue; attention should also be paid to balancing work load and income level. Lastly, organizational change is a positive predictor of change fatigue, which is similar to the results reported in previous studies (10, 38). Nurses who were exposed to frequent or continuous organizational change had higher odds of being in the moderate or high change fatigue groups than in the low change fatigue group. Experienced frequent or continuous organizational changes were 5.054 times more likely to be in the ‘High change fatigue group’ and 2.780 times more likely to be in the ‘Moderate change fatigue group’. This posits that repeated and continuous organizational changes disrupt nurses’ established work routines, increase their work burden, and reduce their sense of control over work, all of which contribute to the development of change fatigue (39). This finding emphasizes that reducing the frequency and intensity of unnecessary organizational changes, or optimizing the pace and mode of change implementation, is crucial for preventing change fatigue among nurses.

### Associations between change fatigue profiles and nurses’ work engagement

After adjusting for years of service, hospital grade, and change exposure, the adjusted BCH analyses revealed significant differences in work engagement and its three dimensions (vigor, dedication, absorption) across the three latent change fatigue profiles (all *p* < 0.001). Pairwise comparisons further demonstrated that nurses in the ‘Low change fatigue group’ scored significantly higher in overall work engagement and three subdimensions than those in the moderate and high change fatigue groups. Notably, however, no significant differences emerged between the moderate and high change fatigue groups on any indicator of work engagement. Specifically, nurses in the ‘Low change fatigue group’ exhibited the highest levels of vigor (16.68), dedication (17.13), and absorption (16.94), reflecting stronger psychological energy, stronger identification with work value, and deeper concentration on nursing tasks (40). However, both moderate and high levels of change fatigue corresponded to substantially lower scores across all components of work engagement. This pattern is highly consistent with COR theory and supports the existence of a threshold effect of change fatigue on work engagement. According to COR theory, individuals strive to protect and replenish personal resources; once resource loss exceeds a critical threshold, further increments in strain do not produce additional linear declines in adaptive outcomes. In the present study, the moderate change fatigue group may already represent a point at which nurses’ emotional, cognitive, and physical resources have been sufficiently depleted. Once this threshold is crossed, even higher levels of change fatigue do not further erode work engagement, as motivational and energetic resources have already reached a floor level. Prolonged adaptation to frequent changes triggers cumulative resource loss, which directly undermines nurses’ vitality at work, weakens their sense of occupational dedication, and reduces their ability to maintain immersive engagement in clinical duties (41). This finding highlights the importance of early identification and stratified intervention: nurses with even moderate change fatigue require timely support to prevent irreversible declines in work motivation and clinical performance. Furthermore, nursing managers should adopt targeted strategies—such as reasonable workload allocation to restore vigor, transparent change communication to reinforce dedication, and optimized working environments to facilitate absorption—may effectively buffer the detrimental effects of change fatigue.

## Limitations

Despite its contributions, this study has certain limitations. First, this study adopted a cross-sectional design, which only reflects variables at a single time point and thus cannot establish causality. Specifically, the cross-sectional nature makes it impossible to determine the temporal and causal relationship between change fatigue and work engagement. To address this, future longitudinal studies (e.g., cross-lagged panel models, cohort studies) are needed to further confirm their temporal and causal relationship. Second, the convenience sampling method may restrict the sample’s representativeness. This study was conducted exclusively in Ningxia Hui Autonomous Region, with geographical limitations that require caution when generalizing results to other regions of China. Future study should employ randomized, stratified sampling across multiple centers. Besides, nurses with <1 year of clinical experience were excluded, so the change fatigue characteristics of this early-career group are not captured. Furthermore, key variables including specific management characteristics of organizational change and nurse psychological resources were not included, limiting the explanatory power of the model. Finally, the assessment of change fatigue still relies on generic management-oriented scales. Future research should develop and validate instruments specific to different types of change in nursing practice.

## Conclusion

Clinical nurses in this study exhibited three distinct change fatigue profiles, highlighting substantial heterogeneity. Years of service, hospital grade, monthly income and change exposure were key demographic predictors of profile membership. The study demonstrates that different change fatigue profiles were significantly associated with work engagement of clinical nurses, suggesting that reducing nurses’ change fatigue is essential for improving their work engagement.

## Data Availability

The raw data supporting the conclusions of this article will be made available by the authors, without undue reservation.
